# Inflammatory response to the ischaemia–reperfusion insult in the liver after major tissue trauma

**DOI:** 10.1007/s00068-022-02026-6

**Published:** 2022-07-14

**Authors:** Yang Li, Annette Palmer, Ludmila Lupu, Markus Huber-Lang

**Affiliations:** Institute for Clinical and Experimental Trauma Immunology (ITI), University Hospital Ulm, Helmholtzstr. 8/1, 89081 Ulm, Germany

**Keywords:** Polytrauma, Inflammatory response, Liver, Organ crosstalk, Ischemia-reperfusion injury, Immunological microenvironment

## Abstract

**Background:**

Polytrauma is often accompanied by ischaemia–reperfusion injury to tissues and organs, and the resulting series of immune inflammatory reactions are a major cause of death in patients. The liver is one of the largest organs in the body, a characteristic that makes it the most vulnerable organ after multiple injuries. In addition, the liver is an important digestive organ that secretes a variety of inflammatory mediators involved in local as well as systemic immune inflammatory responses. Therefore, this review considers the main features of post-traumatic liver injury, focusing on the immuno-pathophysiological changes, the interactions between liver organs, and the principles of treatment deduced.

**Methods:**

We focus on the local as well as systemic immune response involving the liver after multiple injuries, with emphasis on the pathophysiological mechanisms.

**Results:**

An overview of the mechanisms underlying the pathophysiology of local as well as systemic immune responses involving the liver after multiple injuries, the latest research findings, and the current mainstream therapeutic approaches.

**Conclusion:**

Cross-reactivity between various organs and cascade amplification effects are among the main causes of systemic immune inflammatory responses after multiple injuries. For the time being, the pathophysiological mechanisms underlying this interaction remain unclear. Future work will continue to focus on identifying potential signalling pathways as well as target genes and intervening at the right time points to prevent more severe immune inflammatory responses and promote better and faster recovery of the patient.

## Introduction

Despite major improvements in resuscitation and intensive care, trauma remains the leading cause of mortality among those aged under 45 years [1–4]. Although the liver is to some extent protected against external mechanic trauma vectors due to its partial location behind the costal arch, liver is the most vulnerable organ in abdominal trauma [5], where hepatic lesions occur in 66% of cases [6]. Liver trauma remains a clinical challenge for anaesthesiologists and surgeons who have to carefully balance their decision of surgical intervention or non-operative management, for example, depending on the haemodynamic stability of the patient [7]. The liver is a central metabolic organ and is pivotal for both, molecular damage clearance and regenerative processes. Therefore, trauma-induced impairment of the liver function affects the post-traumatic recovery even independently of other additionally damaged organs [8–10]. Because the liver also represents a central hub for the organ crosstalk, it is important to understand the factors that leave trauma patients vulnerable to post-traumatic liver injury and define active measures to prevent additional hepatic damage after trauma. With improved diagnostics and treatment strategies, overall mortality from liver trauma has been reduced. Depending on the injury type and grade, the current mortality rate for these patients is about 10% [11]. The proposed management concepts for liver trauma have been greatly debated for more than two decades. However, a paradigm shift appears to favour non-surgical treatment or damage control surgery, such as liver packing in the case of severe liver trauma [6].

Regarding the complex underlying pathophysiological mechanisms, it is currently suggested that the complex mechanism of post-traumatic liver dysfunction and damage are caused by a variety of factors, including ischaemia and reperfusion injury (IRI), local hepatic and systemic inflammation, endotoxaemia, oxygen radical, cellular apoptosis, and necrosis [12, 13]. A comprehensive understanding of the diverse hepatic responses after trauma appears necessary to rationally address and prevent resulting clinical problems as a consequence of direct or indirect liver trauma.

Therefore, this review considers the main characteristics of liver injury after trauma with a focus on immuno-pathophysiological changes, hepatic organ crosstalk, and deduced treatment principles.

## Hepatic response to major trauma

### Pathophysiological changes

Following major liver trauma or polytrauma, blood loss and the development of shock remain a clinical challenge. Indeed, hepatic failure occurs in 5–10% of patients with polytrauma or haemorrhagic shock [14]. In such cases, the liver function becomes impaired for multiple reasons: due to macro- and micro-perfusion disturbances and associated hypoxic conditions, barrier failure, cellular apoptosis, necroptosis, and necrosis can occur, as typically detected in liver IRI, but also after direct liver trauma [15, 16]. Therefore, assessment and monitoring of the liver function after trauma appear essential. This can be accomplished by the measurement of concentrations of the liver transaminases alanine aminotransferase (AST) and aspartate transaminase (ALT), γ-glutamyl transferase (GGT), alkaline phosphatase (AP), and liver-type fatty acid-binding protein (L-FABP) 1. An acute elevation of AST and ALT blood concentrations to at least 20 times higher than normal can be observed in a shock liver, also known as ischaemic hepatitis [17]. By contrast, in the rare but severe complication of a post-traumatic sclerosing cholangitis, ALT and AST do not increase [18]. Both, AST and ALT were initially defined as markers of liver cell damage [19]. Their appearance in the circulation was explained as passive leakage due to damaged and necrotic hepatocytes. Currently, ALT and AST are increasingly considered as indicators of “hepatic metabolic activity” [20]. AST is widely present in the organism, not only in the cytoplasm and mitochondria of hepatocytes, but, for example, in cardiac and skeletal muscle, brain, and red blood cells. Therefore, it lacks specificity as a marker of liver injury. In humans, ALT1 is found in considerably high concentrations in hepatocytes (particularly in the cytoplasm), while ALT2 is expressed at high levels in fat tissue, kidneys, and brain. Therefore, the ALT1 concentration better reflects hepatocyte damage compared to AST2 [21]. Following major trauma, the development of hepatic failure was shown to be accompanied by significantly elevated AP and GGT concentrations early post-trauma, while the blood concentration of the transaminases remained close to normal but slightly increased at later stages [14]. AP is increased when the tubular membrane of the hepatocyte is disrupted, resulting in the transfer of the tubular membrane to the basal surface of the hepatocyte and subsequent leakage into the serum [22]. GGT is an enzyme that catalyses the transfer of the γ-glutamine moiety of peptides found in the membranes of many tissue cells. It is present in the liver in the membranes of biliary epithelial cells and the apical hepatocytes [23]. When hepatocytes are damaged, GGT is a sensitive indicator for the presence of liver damage by lysing and releasing membrane-bound GGT into the blood. However, many non-hepatic diseases can also cause systemically elevated GGT; thus, its primary use is to confirm whether elevated AP levels are of hepatic origin [24]. L-FABP is a soluble protein found in large quantities in the cytoplasm of hepatocytes and in proximal tubular epithelial cells in the kidney [25–27]. Changes in its levels were previously thought to be associated with liver diseases, including cirrhosis, hepatitis, and hepatocellular carcinoma, and appear to be a possible predictor for survival in chronic liver diseases [27, 28]. Serum L-FABP concentrations can also be applied to assess the amount of hepatocellular damage caused by liver surgery and to detect post-hypoxic tissue damage [29]. Following abdominal trauma, L-FABP appears also as an early marker for acute kidney injury [30].

All IRI events after liver trauma play a central role. IRI is also a common pathophysiological process after polytrauma, haemorrhagic shock, and major liver surgery (including liver transplantation) [31]. Ischaemic conditions with ATP reduction and glycogen consumption mainly in the hepatocytes derive from the consequent lack of sufficient oxygen availability. During a subsequent reperfusion phase, oxygen-induced systemic and mitochondrial reactive oxygen species (ROS) production can exacerbate liver damage. Ischaemia-induced cell dysfunction and death result also in the generation of damage-associated molecular patterns (DAMPs), including histones, mitochondrial DNA, and High-Mobility-Group-Protein Box 1 (HMGB-1), and of inflammatory mediators, including interleukin (IL)-1β and IL-6. The interplay of these hepatic and systemic factors contributes to the activation of the “hepatic” immune system, by activation of non-parenchymal liver cells, including neutrophils, Kupffer cells, dendritic cells, natural killer cells (NK cells), and T cells [32–34]. IRI induces hepatic generation of chemokines and chemoattractants (e.g., complement activation products), which in turn recruit more peripheral immune cells from the circulation to the liver. The immigrated cells not only aid clearance of damaged and infected cells, but can also cause host damage and in consequence exacerbate IRI, reflecting a “vicious circle” of liver damage [35, 36]. In view of the different types and mechanisms of liver cell damage after IRI, liver IRI is classified into two types: (1) warm IRI, caused by liver cell damage at body temperature, mainly occurring after trauma and during haemorrhagic shock, which can lead to liver and multiple-organ dysfunction; and (2) cold IRI, which occurs outside the body at lower temperature during the period of liver preservation (for transplantation), which mainly causes hepatic sinusoidal endothelial cell damage and microcirculation disorders [35, 37, 38]. In addition to the metabolic changes of glycogen consumption, hypoxia, and ATP depletion, the inflammatory immune response can induce direct or indirect cytotoxic mechanisms [35]. Overall, liver IRI appears to result in "holistic" consequences that affect the function of many remote organs, not only of the lungs and kidneys, but also of the intestine, adrenal gland, brain, and other organs [39]. Therefore, therapeutic limitation of liver IRI represents an important topic in clinical and experimental trauma research.

#### Intracellular calcium overload

At present, the exact mechanisms of liver IRI remain unclear [40], but several studies suggested that it is associated with calcium-ion overload and ROS generation [41]. There is evidence that the intracellular Ca^2+^ concentration is a critical factor in hepatic IRI [42]. In a physiologic environment, the intracellular Ca^2+^ concentration is maintained at a relatively low level by mainly three mechanisms: cell membrane selective permeability, ion pumps, and the endoplasmic reticulum. When hepatic IRI occurs, intracellular ATP decreases, resulting in reduced calcium-dependent sodium–potassium pump activity, affecting intracellular calcium-ion transfer to the extracellular space. Consequently, Ca^2+^ released from the endoplasmic reticulum further exacerbates Ca^2+^ accumulation in the cells [43]. Moreover, mitochondria are also subject to Ca^2+^ overload during liver IRI [44, 45]: ischaemia lowered the mitochondrial membrane potential differences, and the transfer of Ca^2+^ to the mitochondrial membrane eventually caused Ca^2+^ overload in this membrane [43, 45]. In vitro simulation of IRI in rat hepatocytes revealed that reperfusion-induced cell death was accompanied by Ca^2+^-dependent mitochondrial ROS formation, which caused mitochondrial permeability transition [46]. In turn, these mitochondrial changes can promote apoptotic events.

#### Oxidative stress response

Liver IRI damage caused by traumatic or other conditions manifests as a sterile inflammatory response marked by ROS overproduction and associated activation of the innate immune system [34, 47, 48]. Although liver epithelial cells can also directly produce ROS during IRI challenge, Kupffer cells are their primary source. At the later and final stages, aggregated and activated neutrophils are considered the primary ROS generators [49]. Numerous enzyme systems can produce ROS in mammalian cells, of which four enzyme systems dominate, which are nicotinamide adenine dinucleotide phosphate (NADPH) oxidase, xanthine oxidase, nitric oxide (NO) uncoupling synthetase, and the mitochondrial electron transport chain. Cascading increases the interaction between these four enzymes, hence intensifying ROS generation and oxidative stress upon stimulation [50]. When migrated neutrophils accumulate in the liver, the CD11b/CD18 on their cells bind directly to intercellular cell adhesion molecule-1 (ICAM-1) on liver cells, which in turn activates NADPH oxidase in neutrophils [51–53], finally resulting in superoxide anion generation. The superoxide dismutase catalyses the reduction of superoxide anion into hydrogen peroxide and then to hydroxyl free radicals [54]. However, it is well established that high levels of ROS cause an imbalance in the body's oxidation and antioxidant systems, leading to irreversible cell damage and cell death not only in the liver but also in remote organs.

### Inflammatory response

Severe tissue injury, for example, after polytrauma with exposure of the patient to DAMPs and hypoxic micromilieus, leads to a local and systemic sterile inflammatory response with the release of multiple chemokines and cytokines [36]. In the liver, IRI is a classical and widely accepted example of sterile liver inflammation [55–58], which includes hepatocytes, neutrophil recruitment and activation, Kupffer cell activation, inflammatory cytokine release, complement activation products, and cytotoxic mediators. Most of these DAMPs and inflammatory mediators are sensed by corresponding pattern recognition receptors (PRRs) [55], which are chemokine and complement receptors on various liver cells that translate the danger signals to an intracellular pathway and cellular defence response [36].

#### Hepatocytes

Multiple DAMPs released by traumatised or stressed liver cells, including HMGB1, ATP, mitochondrial DNA, nuclear DNA fragments, heat-shock proteins, and bile acids [59], bind to PRRs such as receptors for advanced glycation end-products, P2X7R, and Toll-like receptors (TLRs) in the cytoplasm or on cell surfaces [44, 60]. Hepatocytes express a spectrum of TLRs, which mount a potent immune response to DAMPs and other early alarmins [61, 62]. Hepatocytes express TLR2, 3, 4, and 5, while Kupffer cells possess TLR2, 3, 4, and 9 on their surfaces [63]. Some studies suggested that TLR4 functions as an immune surveillance receptor that may exacerbate tissue damage during IRI by enhancing the inflammatory reaction [64, 65]. Of note, although minimal amounts of hepatocyte-derived ROS do not appear to cause cell damage, they can, however, induce HMGB1 release. In turn, hepatocyte-derived HMGB1 can bind to TLR4 on the Kupffer cells’ surface, inducing an inflammatory response and thereby generating larger amounts of ROS [49] which may create a vicious circle.

#### Kupffer cells

**Fig. 1:**
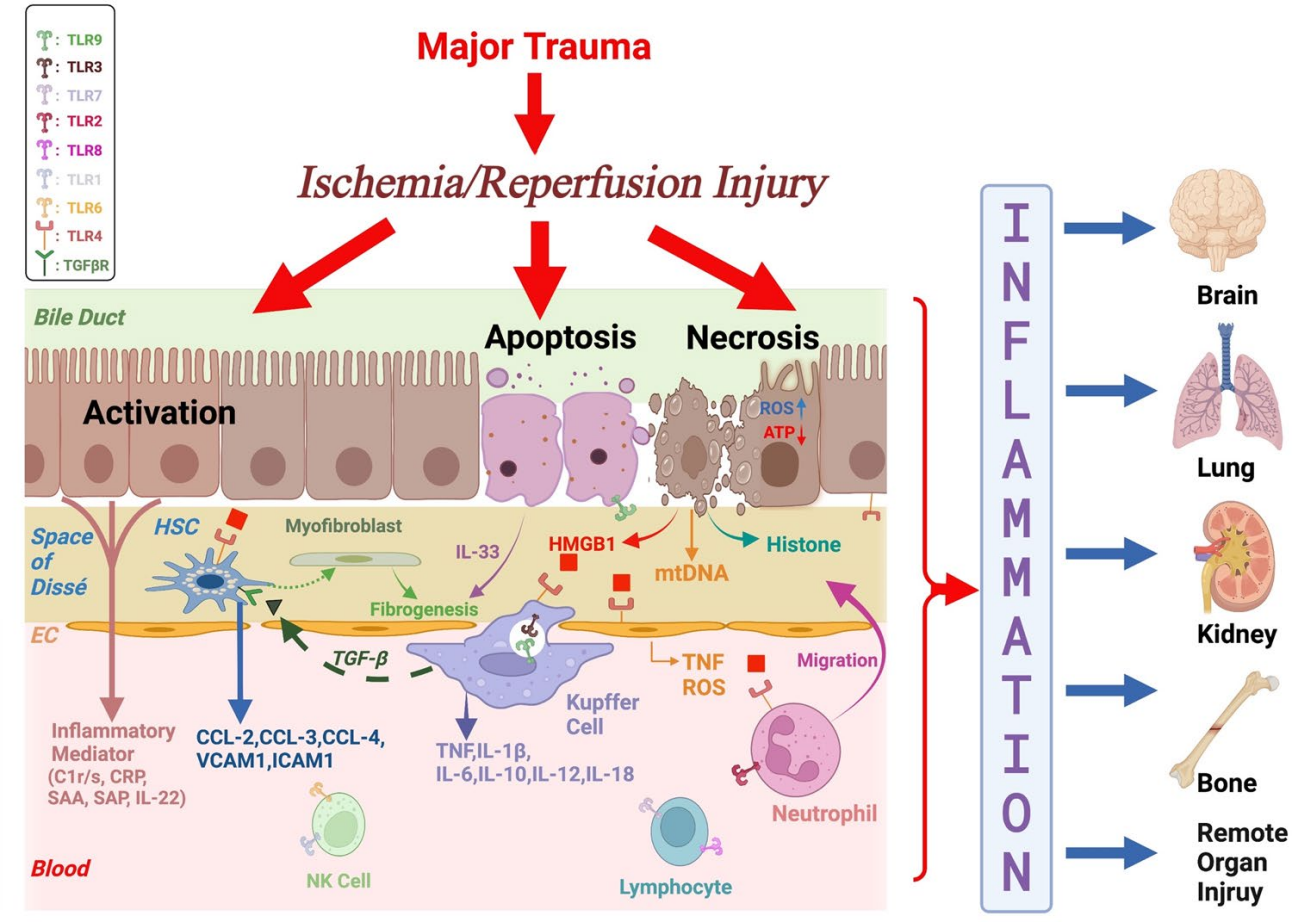
Polytrauma-associated IRI induces an inflammatory response. Polytrauma frequently causes ischaemia/reperfusion of tissues and organs, which in turn results in hepatocyte injury. The damaged hepatocytes become dysfunctional through the activation of KCs or HSCs by various inflammatory factors, including HMGB1, which further generate an inflammatory cascade amplification that acts back on the hepatocytes, causing them to become dysfunctional due to sterile inflammation [81–83]. The inflammatory factors (such as TNF) released into the bloodstream not only act on the liver itself, but also on the brain, lung, kidney, bone, and other distal organ tissues, thus triggering various local immune responses and systemic inflammatory responses. KC: Kupffer Cell; EC: Endothelial cell; HSC: Hepatic Stellate Cell; SAP: Serum Amyloid protein P

Kupffer cells (KCs) in the liver as the largest population of resident macrophages in the body are required for an efficient inflammatory response [66]. KCs are mainly responsible for phagocytosing and clearing tissue debris after trauma. However, in the early stage of reperfusion, activated KCs produce and release ROS and proinflammatory cytokines, including tumour necrosis factor (TNF)-α, IL-1β, IL-2, IL-6, IL-10, IL-12, and IL-18 [49, 67, 68] (Fig. 1). The association between TNF-α and the inflammatory cascade appears crucial for the development of liver damage. TNF-α activates epithelial neutrophil-activating protein-78, nuclear factor-κB (NF-κB), and mitogen-activated protein kinase (MAPK) through binding to corresponding hepatocyte surface receptors, which can directly lead to liver injury [44]. In addition, TNF-α originating mainly from Kupffer Cells can also upregulate the expression of ICAM-1, vascular cell adhesion molecule (VCAM) 1, and adhesion molecules, such as P-selectin, and thereby contribute to inflammatory cell recruitment [69]. In addition to other non-parenchymal cells, KCs also actively secrete HMGB1 as a DNA-binding protein and strong DAMP. Additionally, HMGB1 can be passively released by necrotic KCs and hepatocytes and, irrespective of the release mechanism, induce a strong inflammatory signal [70]. When the liver is subjected to warm ischaemia (after trauma), the HMGB1 level increases and remains elevated for a minimum of 24 h [31]. Overall, activated KCs cause damage to hepatocytes and sinusoidal endothelial cells, eventually leading to hepatocyte necrosis [71].

#### Hepatic stellate cells

Hepatic stellate cells (HSC) or Ito cells prevalent in the space of Dissé express TLR2, 4, and 9. DAMP-driven HSC activation is an important step towards inducing liver fibrosis, whereby HSCs differentiate into myofibroblasts. Furthermore, DAMP sensing by HSCs also induce chemoattractant release, including chemokine (C–C motif) ligand 2, 3, and 4, in addition to the increase of adhesion molecules like VCAM1 or ICAM1 [72]. Thereby, further inflammatory cells can be recruited and the proinflammatory and profibrotic process may progress. However, the exact role of HSCs after tissue trauma requires further clarification.

#### Neutrophils

Following trauma and ischaemia, neutrophils migrate to the liver and can not only clear tissue debris but also cause damage to liver cells via different pathways [44]. The main activators of neutrophils post-trauma are circulating DAMPs originating from damaged mitochondria and anaphylatoxins generated by complement activation [73, 74]. As a consequence of neutrophil activation, these cells belonging to the “first line of defence” accumulate in the liver and increase their levels of CD11a/CD18 (LFA-1) and CD11b/CD18 (Mac-1), which causes adherence to ICAMs, which are increasingly expressed on liver endothelial cells after trauma. Neutrophils migrate along the gradients of chemoattractant, like anaphylatoxins and macrophage inflammatory protein-2(MIP-2) [57], through the hepatic sinusoids and accumulate in the interstitium of liver tissues. ROS generation by the emigrated neutrophils exacerbates liver tissue damage [75–77]. The release of Kupffer cell-inherent proinflammatory chemokines and cytokines can also promote and amplify the neutrophil-mediated inflammatory response [78]. In the later phase of IRI, the emission of various proteases by neutrophils, including collagenases, elastase, and cathepsin G, can cause further cellular liver damage [79, 80] (Fig. 1).

## Liver as a major driver of the inflammatory response

When the liver experiences ischaemia and reperfusion caused by trauma and/or haemorrhagic shock, direct or indirect liver cell damage can occur as described above. The acute phase of IRI in the setting of liver transplantation is characterised by a lack of blood perfusion and oxygenation, which is currently considered as a highly regulated action of the innate and adaptive immunity [84]. It is well documented in the case of multiple injuries that inflammation in the early phase of hepatic IRI is mainly mediated by Kupffer cells and normally occurs rather early (within two hours) after trauma-associated reperfusion. During this period, the pleiotropic cytokine tumour necrosis factor among others is involved as a major proinflammatory factor [42].

### TNF as an important inflammatory contributor to liver damage

Apoptotic cell death in the ischaemic liver is mainly induced by TNF [85], which can be produced by various cells during the inflammatory response-induced apoptosis in liver cells results in NF-κB activation, ultimately altering cysteine and aspartic acid mediated by perforin and granzyme B and/or Fas–Fas ligand complex [86]. In addition, protease activation leads to leukocyte chemotaxis, neutrophil activation, ROS production, mitochondrial toxicity, and apoptosis [61, 87, 88]. Recent research has demonstrated that after haemorrhagic shock-induced liver IRI, the damaging effect of TNF on the liver was mainly based on the following points: (i) During liver IRI, TNF alters Kupffer cell function via autocrine activation, which results in excessive ROS generation and lipid peroxidation, thereby exacerbating neutrophil infiltration and hepatocyte damage [89, 90]; (ii) TNF can participate in pathological processes by producing lipid and other peptide mediators such as prostaglandin [91]; (iii) TNF can activate neutrophils and increase their adhesion to hepatic endothelial cells, leading to the release of various proteolytic enzymes and other inflammatory mediators, and thereby to the constriction of hepatic arteries and an increase in the permeability of liver sinusoids, all of which finally disrupt the barrier and cause liver cell damage [92].

### Complement activation augments the inflammatory liver response

**Fig. 2:**
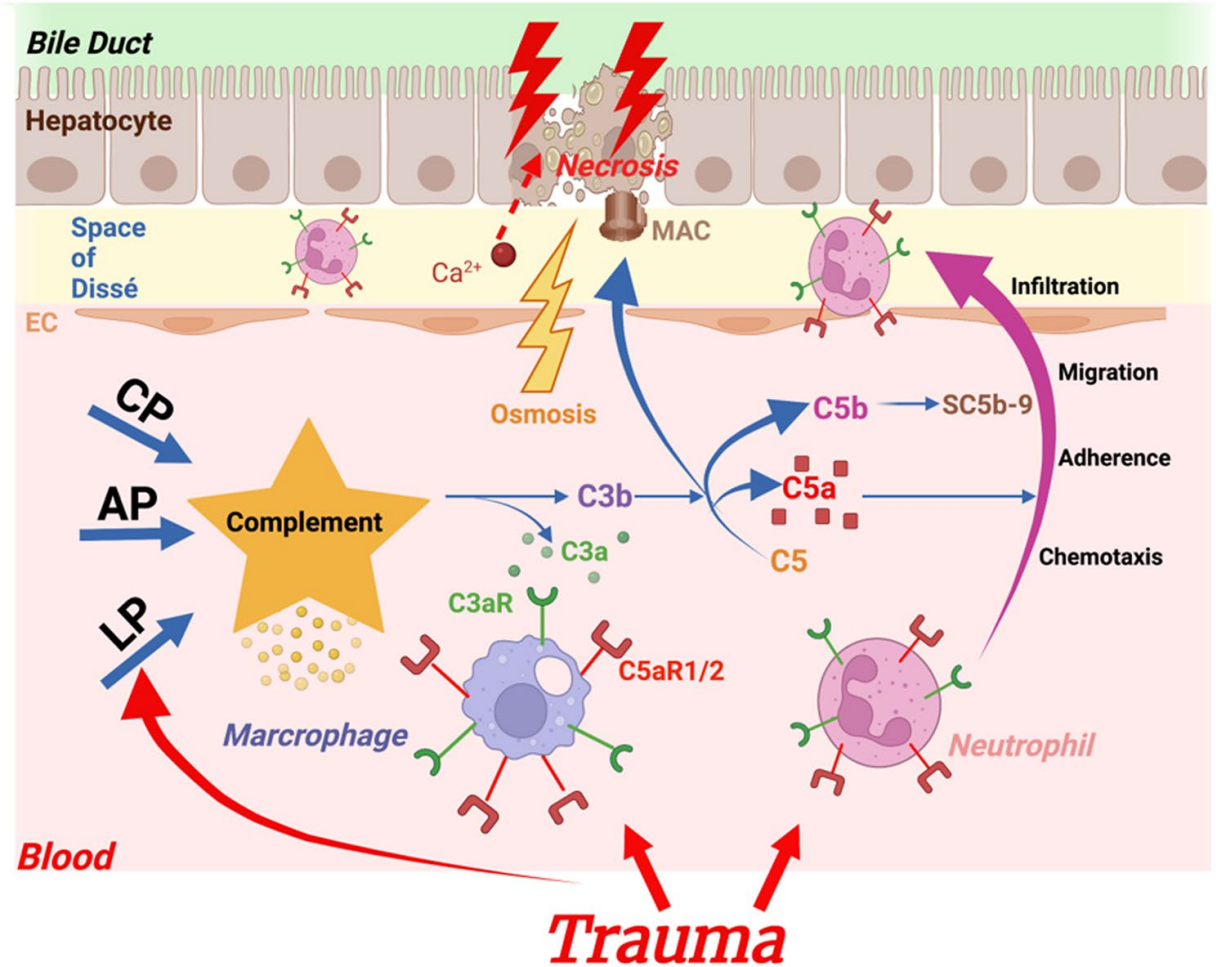
Effects of the complement system on trauma-associated liver damage. Trauma-induced activation of the classical, alternative, and LP pathways results in the generation of the complement anaphylatoxins C3a and C5a. These complement activation products can bind to neutrophils, resulting in indirect damage to liver cells and the formation of the MAC. The MAC causes calcium ions to flow into cells in large quantities, increasing in-cell osmotic pressure and eventually causing cell lysis and death. MAC: Membrane Attack Complex; CP: Classical Pathway; AP: Alternative Pathway; LP: Lectin Pathway; sC5-9: soluble C5b-9

As a major source of most of the plasma complement factors, the liver inherently plays an important role in the complement system and its interactions. Complement as a crucial component of the innate but also the adaptive immune response is typically activated by the binding of circulating recognition molecules like complement component 1q to corresponding molecular patterns [93]. Upon exposure to DAMPs and/or PAMPs, the classical, alternative, and lectin pathways become activated and cleave downstream the central complement protein C3. In ischaemic liver, the interaction of the key cleavage products C3a and C5a leads to neutrophil and hepatic endothelial cell activation via their corresponding receptors C3aR and C5aR1 or C5aR2, respectively. Upon exposure to the most potent chemoattractant anaphylatoxin C5a, neutrophils migrate, aggregate, and adhere to liver sinusoidal endothelial cells. The complement anaphylatoxins can also upregulate adhesion molecules, including VCAM-1 and ICAM-1. Thereby, the anaphylatoxins support inflammatory cell recruitment, resulting in liver cell damage, apoptosis, and necrosis [94–96]. C3b is also a C3 cleavage product and functions as an opsonin. It deposits on the cell surface of ischaemic tissue but can also function as an amplifier of the C3 activation loop via the alternative pathway. Further downstream, cleavage of C5 into C5a and C5b enables the formation of the terminal C5b–9 complex, which can be inserted into the membrane as a membrane attack complex (MAC) or released to the fluid phase as sC5b–9 [94, 97, 98] (Fig. 2). The MAC can be directly introduced into the cell surface to produce two-way hydrophilic pores to allow Ca^2+^ influx, resulting in cell osmotic dissolution and death due to an electrolyte–water imbalance and cellular lysis [99]. Besides these detrimental effects of complement activation triggered by trauma and IRI [74], C3a and C5a also play an important role in liver regeneration processes. C3- as well as C5-deficient mice displayed impaired liver regeneration after carbon tetrachloride- or partial hepatectomy-induced liver injury compared with the same injury in wild-type mice [100–102]. Taken together, complement activation appears in liver damage to be rather Janus-faced: it can add to the inflammatory response early after liver injury but may also help in the resolution of damage and in mediating regenerative processes in later phases (Fig. 2).

### Cytokines and chemokines mounting and disseminating the inflammatory response

IL-1β is a key factor for initiating inflammatory responses and frequently acts synergistically with TNF in hepatic IRI [103, 104]. In the early stages of hepatic IRI, IL-1β released by Kupffer Cells [105] causes neutrophil accumulation at sustainable high levels [106]. The additional release of IL-1β by recruited neutrophils leads to a series of proinflammatory molecular events [107]. The resulting reaction is characterised by the development of cellular oedema, as well as cell lysis and necrosis, which are associated with persistent ischaemia and capillary occlusion [108]. Numerous animal studies have demonstrated that in acute liver injury, necrosis-induced cell death results in rapid IL-1α precursor release, followed by IL-1β and IL-18 up-regulation, ultimately resulting in tissue damage via the IL-1R/IL-18R-MyD88 pathway [109, 110]. Upon initiation of hepatic IRI, excessive TNF is produced in Kupffer cells [111], and this induces macrophages to produce large amounts of IL-10, which in turn inhibits NF-κB activation and reduces the overexpression of inflammatory cytokines. These events may reduce the hepatic and systemic inflammatory responses [112]. However, binding of proinflammatory cytokines such as TNF to hepatocytes appears to upregulate TLR mRNA levels [113]. This signalling cascade offers a promising therapeutic target to ameliorate the inflammatory response.

IL-6 is a multifunctional cytokine that can be rapidly produced during the acute phase of the inflammatory response and thus contributes to the host defence directly after trauma. In numerous studies, enhanced IL-6 concentrations in the circulation were detected correlating to the injury severity score and exhibiting some prognostic value [114, 115]. During the initial phase of inflammation, systemic IL-6 reaches the site of the liver through the bloodstream, which in response rapidly produces various acute phase proteins, including C-reactive protein (CRP), serum amyloid A (SAA), fibrinogen, and others [116]. In animal experiments, it was found that rats with genetic IL-6 knockout displayed more severe liver damage after hepatic IRI than the control group [117]. Rapid local and systemic IL-6 production after hepatic IRI also enhanced the activity of T and B lymphocytes and NK cells, and was central in inflammatory crosstalks of the liver [118].

IL-10 is a protein of approximately 18 kDa that can be synthesised by T lymphocytes, B lymphocytes, monocytes, and macrophages. It is a mainly anti-inflammatory cytokine and has been shown to reduce TNF and IL-1 synthesis in vitro and in vivo [119]. It is generally recognised that signal transducer and activator of transcription 3 activation by IL-10 receptors in myeloid-derived cells is a necessary step for the anti-inflammatory effects of IL-10, as demonstrated by an STAT3-deficient mouse model [120]. IL-10 was found to reduce the IRI-induced microcirculatory impairment and oedema in the liver. On a molecular level, IL-10 reduced TNF and MIP-2 expression and blood levels [121]. By contrast, a study on intestinal IRI found that IL-10 resulted in increased tissue damage. This adverse effect may be the result of reduced nitric oxide synthase (NOS)-2 and haem oxygenase (HO)-1 mRNA expression [122]. However, in the liver, the role of IL-10 during hepatic IRI appears rather to be protective. IL-10 plays a supportive role for hepatocyte proliferation and a clear protective role in parenchymal liver injury (IRI-induced) with a reduction in the apoptosis and necrosis rates [53, 123]. Numerous IRI studies in rodents have also shown that animals with genetic IL-10 deletion or animals receiving anti-IL-10 treatment suffered more severe hepatic damage than the corresponding control groups [123, 124]. Therefore, IL-10 appears to be a promising therapeutic mediator in the case of liver injury, which requires further translational investigations.

IL-17 was originally reported by Rouvier et al. as CTLA8 and was subsequently renamed IL-17 (also called IL-17A) [125]. It was first found to be derived from TH17 cells. Later, Natural killer T and Paneth cells were also found to secrete IL-17 [126, 127]. The contribution of IL-17 to post-traumatic immune dysfunction following polytrauma is uncertain [128]. During liver IRI, damaged hepatocytes induce KCs to release large amounts of cytokines, including IL-1β and IL-6, which in turn promote the generation of significant amounts of IL-17 by TH17. IL-17 is mainly found in neutrophils in the liver, and vice versa, IL-17 promotes the recruitment of neutrophils towards the inflammation site, thereby amplifying hepatic IRI [129]. One research group proposed that systemic IL-17 has no predictive value for the prognosis after polytrauma. Although systemic IL-17 levels were elevated in a minority of patients with multiple injuries, they speculated that this may be related to individual variance and susceptibility [128]. Therefore, the value of IL-17 to assess a patient’s course and prognosis after multiple injuries requires further clinical evaluation.

### Nuclear factor-kappa B (NF-κB) involvement in the liver cell response

Numerous studies have demonstrated that the NF-κB signalling pathway is one of the main mechanisms of liver IRI damage [130, 131]. It has been shown that NF-ΚB is regulated by many factors, including inducible NOS (iNOS), chemokines (C-X-C motif) ligand 78, and ICAM-1 [78, 132]. NF-κB-inducing kinase binds R-associated factor 2 in the tumour necrosis factor receptor complex in response to stimulants and phosphorylates the I-kappa-B (IκB) kinase complex (IκK) via the R signalling pathway. The activated IκK directly phosphorylates the IκB-specific site, Ser/Tyr, leading to its degradation and dissociation from NF-κB, thereby allowing the NF-κB p50/p65 dimer to enter the nucleus and initiate a series of subsequent transmembrane signal transductions [133]. During hepatic IRI, the binding activity of NF-κB to its specific regulatory gene sequences was found to be highest at 2–3 h after hepatic ischaemia–reperfusion, suggesting a direct relationship between this NF-κB-specific regulatory gene sequence-binding activity and the extent of injury [121]. Furthermore, it appears that NF-κB binding activity in liver tissue after IRI is temporally phased, together with increased TNF and ICAM-mRNA expression [134]. Takahashi et al. demonstrated that the NF-κB p65/p50 dimer is activated early and rapidly expressed at high levels, initiating inflammatory responses in liver tissue [135–137]. During liver IRI, it has been shown that NF-κB activation in hepatocytes rather represents a protective mechanism, which can be enhanced by ischaemic hypothermia [138, 139]. In agreement with this, pre-treatment with NF-κB ligand receptor activator attenuated IRI in the rat liver. This protective mechanism was associated with activation of the NF-κB signalling pathway in hepatocytes [140]. However, the exact role of NF-κB in trauma-induced direct and indirect hepatic injury beyond IRI remains to be defined.

## Liver as the primarily damaged organ

Kupffer cells are the main source of cytokines and inflammatory mediators, and are involved in the amplification of the systemic inflammation as suggested in a setting of liver transplantation-induced IRI [141]. The DAMPs and associated inflammatory mediators signal via PRRs such as TLR4 (see above), eventually resulting in development of a systemic inflammatory response syndrome (SIRS), which ultimately involves multiple organs, including the brain, lungs, and kidneys. If the remote organ response becomes dysfunctional, this is clinically manifested as multiple-organ dysfunction syndrome [142, 143]. Among many critical organs, the liver as the largest metabolic organ in the body is the central target organ for severe immuno-pathophysiological damage [144]. Vice versa, after liver injury, the transcriptome of extrahepatic organs is dramatically altered, suggesting that serum metabolite-mediated crosstalking networks between the liver and extrahepatic organs are very important [8].

### Liver–organ crosstalk

#### Brain–liver-axis hepatic IRI causes brain injury

In a hepatic IRI rat model, long-term cognitive function was impaired [145]. In mice, liver IRI led to short-term dimensional cognitive deficits, which was time-dependent: the longer the time elapsed after the onset of ischaemia, the poorer was the cognitive function, which may be associated with the large amount of harmful substances produced at the time of IRI initiation, including ROS and inflammatory factors [146]. NF-κB can exacerbate liver ischaemia and reperfusion damage with remote effects of disrupting neurological development and brain damage repair processes, thereby impairing cognitive functions. In particular, the expression of inflammatory factors induced by NF-κB in brain tissue may result in short-term cognitive impairment [147]. Zheng et al. found that E2f transcription factor 8 (E2f8) plays an important role in the intersectional network between hepatic IRI and brain injury. In the underlying experimental design of two different surgical liver injury models of liver resection (LR) and bile duct ligation (BDL), principal component analyses revealed that the E2f8 transcription factor levels were upregulated in the LR and BDL group [8]. E2f8 is an essential transcription factor for angiogenesis, lymph angiogenesis, embryonic development [148], and adult neuronal cell differentiation [149]. Therefore, the authors concluded that further investigations are required to determine whether E2f8 plays an important role in the development of hyperammonaemia and how to regulate E2f8 in the brain during acute liver injury. Nevertheless, at present, the particular molecular mechanisms underlying brain damage after liver ischaemia–reperfusion and even more so in the setting of polytrauma remain unclear.

#### Lung–liver neighbourhood

Essential events in lung IRI are the formation of ROS and reactive nitrogen species (RNS), complement activation, and the generation of inflammatory mediators such as TNF [150]. The mitochondrial respiratory chain not only provides ATP but also produces by-products such as ROS. After hepatic IRI, KCs release large amounts of ROS that lead to cell death through the opening of voltage-dependent anion channels1 and alter the opening of the permeability transition pore complex in the presence of adenine nucleotide transporter enzymes [151, 152]. Both RNS and NO can severely damage cells in a similar manner to ROS. RNS increase the opening capacity of the permeability transition pore complex by covalently modifying many proteins, including mitochondrial complex IV and glycerol-3-phosphate dehydrogenase, inducing cell death and subsequent pulmonary damage [153, 154]. An increase in both TNF and IL-1β has been detected 2–7 days after BDL surgery [155]. Accordingly, monitoring of inflammatory mediators, including the IL-1β, IL-6, and IL-10 levels, in patients after major trauma (including liver transplantation) appears rational to minimise the risk of missing hidden damage to the liver and remote organs. However, respective clinical studies are currently lacking.

#### Hepato-renal crosstalk

The liver–kidney axis represents a clinically established important cross-talk. Hepatic IRI is an important contributor to acute kidney injury (AKI) and trauma-related AKI [156] as well as reduced recipient survival and chronic kidney disease in liver transplant recipients [157]. The onset of hepatic IRI frequently causes AKI characterised by a decline in urine output and enhanced retention parameters based on a decrease in the glomerular filtration rate. Histologically, the hepatic injury can result in early renal endothelial cell apoptosis, proximal tubular inflammation (by cytokine and neutrophil infiltration) and necrosis, intrarenal vascular permeability impairment, and renal proximal tubule epithelial filamentous-actin degradation [158]. Interestingly, pre-ischaemic injection of human A_1_ adenosine receptor (huA_1_AR) in the liver is somehow effective in protecting against both hepatic and renal injury modelled by hepatic IRI. The reason for this dual beneficial effect is, however, associated with the selective overprotective nature of the cytoprotective A_1_AR in the kidneys [159]. Other mechanisms have been proposed for an improved hepato-renal axis, such as the downregulation of HO-1, autophagy-related 7, and peroxisome proliferator-activated receptor gamma cofactor 1-alpha through the use of some anti-inflammatory and antioxidant drugs, including sphingosine-1-phosphate. These treatment strategies have also exerted a protective effect against hepatic IRI-induced kidney injury [160, 161].

#### Liver–bone relationship

Fractures to the pelvis, femur, and other major bones can lead to traumatic haemorrhagic shock, resulting in severe liver IRI and subsequent sterile inflammation. The liver is involved in bone formation and repair mechanisms through the regulation of parathyroid hormones (PTH) and the synthesis of growth factors such as insulin-like growth factor (IGF)-I and IGF-II [162, 163]. PTH affects the osteoactive hormone response by inducing liver IL-6 production [164]. It can even metabolise various bone-active molecules, shortening their half-life and affecting their circulating levels in humans [165]. Severe trauma with multiple fractures and subsequent haemorrhagic shock frequently lead to hepatic IRI [166]. The "second (multiple) hit" after trauma can amplify the existing inflammatory state and results in an excessive SIRS, which can lead to symptoms of liver failure [167, 168]. The length of time without treatment after the onset of trauma is thought to be an important factor for the liver and bone healing processes. A clinical study demonstrated a significant increase in hepatic myeloperoxidase activity within 1 h after trauma, a significant increase in liver permeability after 2 h, and a peak in serum AST levels at 3 and 5 h post-trauma [169]. The inflammatory factors released by femoral fractures are cross-linked to those released by hepatic IRI, yet the mechanism regarding the influence of femoral fracture healing remains unclear. The fact that systemic inflammation or excessive local inflammation can compromise fracture healing has been confirmed by numerous studies [8, 170, 171], but the process by which fracture healing is affected remains unclear. Therefore, further clarification is required on how individual inflammatory factors generated particularly in the liver are involved in the inflammatory and regenerative processes of fracture healing.

## Therapeutic approaches

Recent studies have focused on the inhibition of the TLR4-NK-κB signalling pathway to improve hepatic IRI. Inhibition of TLR4-NK-κB signalling by pre-treatment with injected Eucommia polysaccharide, iridoid glucoside aucubin, or cell-free matrix hydrogels attenuated hepatic IRI [172–174]. Huang et al. further found that the inhibition of miR-450b-5p ameliorated hepatic IRI by targeting alpha B-crystallin [175]. Therefore, the role of NF-κB in hepatic IRI remains of major interest for future research. The immunomodulatory regulation of the proinflammatory response of NF-κB and the regulation of the cell cycle and other related biological effects may in principle play a beneficial role. Whether this is true in the clinical real-world setting remains to be evaluated.

Another approach aims to intervene earlier. Inflammasomes are multiprotein complexes that initiate the release of the proinflammatory cytokines IL-1β and IL-18 by activating caspase-1 through sensing danger signal release. Inflammasome activation also contributes to sterile inflammation following hepatic IRI [176]. Several groups have reported that employing caspase activators in animal models of hepatic IRI prevented apoptosis, improved organ function, and increased the survival rate [15]. In addition, numerous immunotherapies have been developed to target specific signalling pathways. A study of Artesunate to reduce liver injury due to haemorrhagic shock (HS) found that Artesunate treatment of HS rats enhanced protein kinase B and endothelial NOS activation, inhibited phosphorylation of glycogen synthase kinase 3β, attenuated activation of NF-κB, and reduced the expression of iNOS, TNF and IL-6 [177]. Clinical translation is feasible here, because Artesunate is already used in the clinic as an approved anti-malaria drug.

Regarding complement targeting, specific C5 complement inhibitors are also used to protect the liver from IRI. A study in mice found that blockade of C5a-mediated responses not only inhibited platelet aggregation in the early stages after the onset of hepatic IRI, but also attenuated infiltrating macrophage/neutrophil activation and hepatocyte apoptosis in the later stages of reperfusion [178]. A clinical study similarly confirmed that the C5a/C5aR1 interaction has an important regulatory capacity on the trauma-induced delayed apoptosis of polymorphonuclear cells. Of note, while C5a significantly inhibited apoptosis in neutrophils, the other anaphylatoxin C3a failed to show similar effects [179]. Therefore, C5a may present a promising target.

Recent research also focused on the therapeutic potential of the activation of the nuclear factor E2-related factor 2, for example, by triterpenoid CDDO-Imidazolide. In IRI, protective effects were identified by limiting the inflammatory response of the liver and hepatocyte cell death [180]. Intrinsic cyclin-dependent kinase 2 (CDK2) expression typically increases after the reperfusion. Inhibition of CDK2 by Roscovitin protected the damaged liver by inhibiting macrophage/neutrophil infiltration into the liver, and suppressed in vitro the TLR 4 signalling pathway by regulating the MAPK pathway in macrophages [181]. However, these promising approaches need future translational evaluation, particularly in the context of trauma.

## Outlook

The liver is the central metabolic organ of the human body; it not only generates and secretes inflammatory factors but also serves as a target organ for their actions. The mechanisms of liver self-repair and regeneration after trauma often entail cell-mediated clearance of dead cells and tissue remodelling regulated by different mediators. Currently, we still do not understand the exact mechanisms of hepatic inflammation, repair, and self-regeneration after the onset of traumatic liver injury. Future studies including clinically relevant trauma modelling (in vitro and in vivo) will help to elucidate underlying molecular mechanisms and evaluate targeted therapeutic approaches in the trauma setting to reduce the risk for secondary liver injury and thus to improve patient outcome post-trauma.
